# Biofunctionalization and Applications of Polymeric Nanofibers in Tissue Engineering and Regenerative Medicine

**DOI:** 10.3390/polym15051202

**Published:** 2023-02-27

**Authors:** Prasanna Phutane, Darshan Telange, Surendra Agrawal, Mahendra Gunde, Kunal Kotkar, Anil Pethe

**Affiliations:** 1 Department of Pharmaceutics, Datta Meghe Institute of Higher Education and Research, Datta Meghe College of Pharmacy, Wardha 442004, MH, India; 2 Department of Pharmaceutical Chemistry, Datta Meghe Institute of Higher Education and Research, Datta Meghe College of Pharmacy, Wardha 442004, MH, India; 3 Department of Pharmacognosy, Datta Meghe Institute of Higher Education and Research, Datta Meghe College of Pharmacy, Wardha 442004, MH, India; 4 Department of Pharmaceutical Quality Assurance, R.C. Patel Institute of Pharmaceutical Education and Research, Shirpur 425405, MH, India

**Keywords:** nanofiber, scaffold, electrospinning, tissue engineering, regenerative medicine

## Abstract

The limited ability of most human tissues to regenerate has necessitated the interventions namely autograft and allograft, both of which carry the limitations of its own. An alternative to such interventions could be the capability to regenerate the tissue in vivo.Regeneration of tissue using the innate capacity of the cells to regenerate is studied under the discipline of tissue engineering and regenerative medicine (TERM). Besides the cells and growth-controlling bioactives, scaffolds play the central role in TERM which is analogous to the role performed by extracellular matrix (ECM) in the vivo. Mimicking the structure of ECM at the nanoscale is one of the critical attributes demonstrated by nanofibers. This unique feature and its customizable structure to befit different types of tissues make nanofibers a competent candidate for tissue engineering. This review discusses broad range of natural and synthetic biodegradable polymers employed to construct nanofibers as well as biofunctionalization of polymers to improve cellular interaction and tissue integration. Amongst the diverse ways to fabricate nanofibers, electrospinning has been discussed in detail along with advances in this technique. Review also presents a discourse on application of nanofibers for a range of tissues, namely neural, vascular, cartilage, bone, dermal and cardiac.

## 1. Introduction

Humans possess the finite ability to re-grow or regenerate tissues, organs or any part of the body after its resection, except some organs such as liver and lungs possess the good capability to regenerate. Bones and smooth muscles have limited ability to regenerate, while others that scarcely regenerate include the cardiac muscle, lens of the eye, skeletal muscle and nerves. Injury to or resection of many such tissues creates the problem of loss of functionality and unpleasant appearance. Autograft and allograft are the currently available treatments for injury or trauma caused to the tissue. But they carry multiple limitations with them, proposing the researchers to look for better alternatives. The tissues having the capability to regenerate themselves will be the best possible answer, which is evidently not possible in humans rightnow.However, human tissues can be assisted for such regeneration. Such regeneration of tissues applying the principles of life sciences and engineering and using the innate capacity of the cells, is studied under the discipline of tissue engineering and regenerative medicine (TERM).

**Figure 1:**
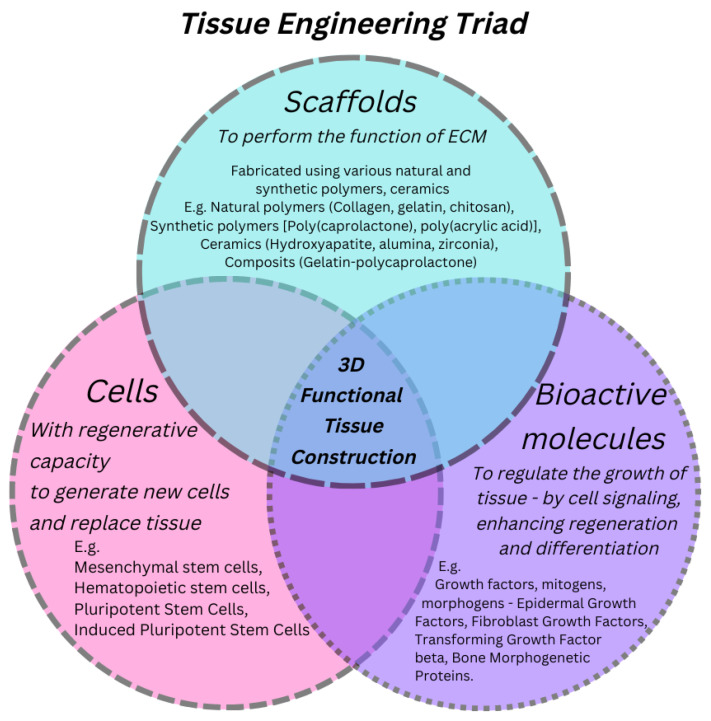
Tissue Engineering Triad.

The key components required for engineering a tissue are regenerative cells, scaffolds and growth-controlling bioactive molecules. These are commonly called as the tissue engineering (TE) triad [1] as shown in Figure 1. Scaffolds have the central role to perform in TERM which is analogous to the role performed by Extracellular Matrix (ECM) in the biological tissues. Scaffold, alike ECM, render structural reinforcement and physical milieu for cells to adhere, multiply, differentiate and migrate. But the scientists are having as enormous variety of choices in scaffolds for TE application. Moreover, mimicking the structure of the ECM at the nanoscale in fabricated scaffold was one of the great limitations in the research area of TE. This limitation was concluded to a great extent by the development of nanofibers. Architecture of ECM consisting of an interwoven fibrous structure in nanoscale range made of an array of multidomain macromolecules inspires the fabrication of scaffolds. Emulation of the structure of ECM to invoke the biological function of the ECM has been one of the central area of research in TE [2].

Nanofibers based systems have been explored for a wide variety of biological [3] as well as non-biological applications [4,5] because of its highly controllable properties. Its biological applications include burn and wound dressing [6,7], facemasks [8], tissue regeneration [9], osteoporosis treatment [10] and drug delivery [11].

## 2. Nanofibers Based Scaffolds in Tissue Engineering

Nanofibers possess some unique features which make it competent candidate for TE application. Some of them are discussed in following paragraphs.

High surface area to volume quotient and consequent high surface energy of nanofibers in comparison to bulk materials results in better attachment of cells, proteins and drug molecules [10]. In comparison to other special kinds of tissue scaffolds such as foam and gel films, fabrication of nanofibers furnishesan opportunity of achieving higher surface area for an equal volume.

High porosity of the scaffolds is preferred to allow for migration, attachment and proliferation of cells, for circulation of oxygen, nutrients and disposal of metabolic byproducts. But an inverse relation has been observed between porosity and tensile strength [12]. Thus, it became difficult for the researchers to achieve mechanical strength, while aiming at high porosity. But nanofibers provide the sufficient mechanical strength, while attaining high porosity. It also showed to possess organized porous architectures and porosity achieved in nanofiber based scaffolds was reported to be more than 90 percent [13]. Nanofibers resemble the porous arrangement of ECM, hence they are favorable for tissue regeneration [10].

Nanofiber commonly demonstrates high aspect ratio, which is a ratio of the length to width of the fiber [9]. High aspect ratio is associated with good tensile strength of the fibrous matrix, due to lengthier nanofibers which impart overall strength to the fibrous matrix [14].

Nanofibers are a peculiar class of material providing biomimetic environment at nanometric level, suitable surface properties and three-dimensional framework on the micrometric level and mechanical performance and physiological acceptability on the macrometric scale [15] Furthermore, many ex-vivo studies on scaffolds, facemasks and wound dressings of nanofibrous origin have established their superiority over their counterparts composed of the same material at micro or macrometric scale [16].

As the attributes of the nanofibers are very sensitive to the properties of the polymer and parameters used in manufacturing techniques, these attributes of the nanofibers can be regulated according to the required application. Thus, flexibility of nanofiber assemblies can be tailored to great magnitude. Some other manageable attributes are diameter of the fiber, flexibility, directional properties, etc. Morphology of nanofibers can be customized to befit various types of tissues or to encapsulate biologically active molecules.In addition, a great range of polymers can be electrospun to serve different applications.

These noteworthy properties make nanofibers idyllic candidate for a broad variety of biomedical and healthcare applications, including TERM.

## 3. Bio-Degradable Polymers

The selection of material is a central consideration in regulating the utility of the nanofibers for TERM. Biocompatibility is the foremost feature of the polymer to consider for its use in the biomedical applications, followed by biodegradability. There are several polymers which are biocompatible but non-biodegradable. Polymers can be generally categorized based on the susceptibility of their chemical backbone to degradation on exposure to water by the process of hydrolysis, as non-biodegradable and biodegradable. For tissues namely bone, tendons, cartilages, ligaments, and blood vessels, mechanical character is a prime factor. For tissue regeneration of such tissues, non-degradable polymers find a use. Non-biodegradable polymers have also been tried in guided TE application such as directing re-growth of tissues. They have been used for orbital reconstruction, facial reconstruction and rhinoplasty [17]. They also find use in ex-vivo guidance of tissue growth. Some examples of non-degradable polymers used are poly(tetrafluroethylene) (PTFE), extended-PTFE, polyurethane, poly(ethylene terephthalate), poly(ethersulfones), etc. Similarly, gels made from non-degradable polymers such as poly(ethyleneglycol), poly(ethylene oxide) (PEO), poly(vinyl alcohol) and Pluronic (block co-polymers of PEO and poly(propyleneoxide)) have been explored in the domain of engineering of scaffolds.

Another category of biomaterials called tissue adhesives possess the adhesive properties and help to stick the non-adhesive scaffold devices in vivo. Examples of such tissue adhesives are fibrin, albumin and cyanoacrylates. But such tissue adhesives can not be utilized as the proper scaffold to regenerate tissue owing to many of its limitations. To conquer these limitations, adhesive tissue engineering scaffolds (ATESs) have been developed. These ATESs can be secured at the site in vivo without the need of gluing or suturing [18].

With the evolution of TE discipline, the focus has been shifted more on ‘functional TE’. There has been a convincing assertion for the use of degradable polymers to fabricate hybrid tissue equivalents. With the overarching need to dissolve the synthetically produced tissue equivalent in situ, in progressive way and in tandem with the process of tissue regeneration, biodegradable polymers came in the spotlight. This review will emphasize primarily on the biodegradable polymers, as it forms the large chunk of the research on TE.

Numerous biodegradable polymers, both natural as well as synthetic, have been utilized in the production of scaffolds based on nanofiber with diverse morphological features. Each class of polymer has its unique set of attributes some of which are expedient for fabrication of scaffolds whereas others are detrimental to overall performance of the scaffold. Table 1 enumerates some of the strengths as well as weaknesses of polymer of natural and synthetic origin for its application in TE. To utilize the strengths of polymers of both natural and synthetic origin, scientists have manufactured composite scaffolds having mechanical properties and bioactivity suitable for regeneration of tissues. Table 2 enlists polymers and polymer composites used to electrospun nanofibers for different TE applications with the results of in vitro/in vivo evaluationtests.

In the following paragraphs, the natural and synthetic polymers employed in fabrication of scaffolds are discussed with their strengths and weaknesses.

### 3.1. Natural Polymers

Polymers of natural origin are biocompatible, biodegradable and show low immunogenicity. They offer the advantage of being similar and many a times identical with the ECM, thus elicits the favorable interaction with the cells. In addition, some possess anti-microbial and anti-inflammatory properties, thus buttressing the course of tissue repair and regeneration. Some of the limitations in fabrication of nanofibers using natural polymers are difficult processing, lowcost effectiveness, poor mechanical properties and precarious outcome. Natural polymers also show variations in its characteristics between different batches and different sources. Insufficient mechanical strength and greater water solubility are the limitations of most of the naturally derived biopolymers used to construct the scaffolds. These limitations are overcome by the way of crosslinking to preserve their architectural cohesion in aqueous medium and to increase mechanical toughness. The issue associated with the crosslinking is the cytotoxicity of the chemicals utilized to crosslink. Enzymatic as well as physical methods have been explored for the crosslinking of the biopolymers along with crosslinking using non-toxic and low-toxic chemicals [30]. But crosslinking using non-toxic chemicals demonstrate the low degree of crosslinking compared to crosslinking with glutaraldehyde and other common toxic chemicals. Some examples of extensively studied natural polymers to manufacture nanofibers for application in TE are collagen, gelatin, alginate, chitosan, hyaluronic acid and silk fibroin.

#### 3.1.1. Collagen

Collagen is the most plentiful protein in humans and animals and is the prime ECM protein which imparts it structural integrity [31]. Thus, it can be inferred that nanofibers made out of collagen will most closely mimic the histological structure of native tissues. Other suitable attributes of the collagen are the induction of very low immunogenic response and its suitability for the regeneration of most body tissues [32]. One of the main deficiencies of the collagen is poor mechanical strength. Scaffolds made out of pure collagen displays inadequate resistance to water and collagenase which results in reduced rigidity to withstand handling while implanting the scaffold [33]. Mechanical strength can be improved via cross-linking, which imparts degradation resistance and increased strength. D-banding observed with the quaternary structure of native collagen type I is pivotal to the mechanical stability of native collagen. Such D-banding is lost in solubilization process during processing to construct scaffolds [34]. Thus, methods which could preserve or recreate D-banding needs to be explored, to overcome the limitation associated with constructs designed out of collagen. Mechanical features can also be improved by deciphering the origins of the unique mechanical attributes of the native collagen fibrils. The mechanical strength of collagen microfibrils originate from the hierarchical structure at the nanometer scale, which, upon application of stress, results in straightening of twisted molecules, followed by stretching at the axis and further molecular uncoiling. Such sequence of deformation mechanisms impart collagen fibrils its’ strength, specifically its great extensibility, strain hardening, and toughness. Usage of pure collagen molecules in its primary structure can not provide the broad range of mechanical functionality necessary for physiological functioning of collagenous tissues [35]. The hierarchical structure of the biological collagen fibrils inspires the fabrication of scaffolds which reproduce dimensional aspects and functionality of the native ECM.

A study developed mineralized nanofibrous composite structure similar to bone with electrospun collagen containing catecholamines and Ca^2+^. Divalent cation induced crosslinking of collagen nanofibers, thus providing constructs with mechanical strength. Further mineralization of construct ammonium carbonate resulted into scaffold with exceptional mechanical strength with Young’s modulus nearing the thresholds of cancellous bone. The scaffolds showed excellent biocompatibility with human fetal osteoblast cells and osteogenic efficiency [19].

#### 3.1.2. Gelatin

Gelatin is partially hydrolyzed form of collagen having shorter chains of amino acids. Even though gelatin is technically a form of collagen, gelatin is less expensive, more readily available, presents a reduced immunological risk, provides improved hydrophilicity and cell adhesion [36]. Dissimilarity in the chemical composition of these two proteins makes them each act very differently. Gelatin nanofibers have demonstrated to be efficacious scaffolds for TE application with good cell adhesion activity. Thermo-responsive property of gelatin aqueous solution is a crucial feature which causes its’ reversible transformation from sol to gel when temperature lowered below its critical solution temperature [37]. But the same becomes the limitation due its gelation at ambient temperature range. In a study to manufacture electrospun gelatin nanofibers, numerous organic solvents have been screened for their potential to preserve gelatin in a sol state at ambient temperature. Fluorinated alcohols as well as acidic organic solvents are observed to impede gelatinizing at room temperature [20]. The thermo-responsive behavior of gelatin can also be used to achieve desirable viscosity of the gelatin solution for nanofiber making.

#### 3.1.3. Alginate

Alginate, also known as algin or alganic acid, has been one of the materials of choice for TE. But the electrospinning technique has affixed a new dimension to this polymeric material. Alginate is a natural polysaccharides, extracted from the cell walls of brown algae [38]. Alginate possesses some exceptional properties, namely high biocompatibility, fairly low immune response, and unique gel-forming capacity. It also shows structural similarity to proteoglycans, which is a crucial element of the ECM [39]. One of the limitations of alginate is its inability to precisely interact with mammalian cells. Thus, the material must be adapted to support cell adhesion. One of the means is attaching of cell adhesive peptides with covalent bonding to the polysaccharide backbone of alginate [40]. Despite the potential of alginate nanofibers, electrospinning trials of pure alginate nanofibers have not been successful. Dense intra- and intermolecular hydrogen bonding in the alginate was reported to pose a challenge in its electrospinning. To produce continual running and uniform nanofibers from the electrospinning of pure alginate solutions whether aqueous or non-aqueous, is an arduous task [41]. But its’ electrospinnability can be improved by mixing it with polymers like polyvinyl alcohol [42] and polyethylene oxide [43]. However, existence of impurities in manufactured scaffold and challenges in bulk production of alginate-based nanofibers are still unresolved challenges for alginate polymer.

In a study, sodium alginate/polycaprolactone core-shell nanofibers were prepared using emulsion electrospinning. Water in oil emulsion was prepared where sodium alginate aqueous solution formed the dispersed phase whereas polycaprolactone in chloroform formed the continuous phase. This core-shell nanofiber was developed to act as promising candidate for incorporating both hydrophilic and hydrophobic bioactive molecules for biomedical application [21].

#### 3.1.4. Chitosan

Chitosan is another polysaccharide that has been extensively examined as a biomaterial for scaffold fabrication for tissue regeneration purpose. Chitosan nanofibers are quite commonly used in the area of TE on account of its’ morphological and chemical analogy with natural ECM, thus being biocompatible and biodegradable. In addition, its antimicrobial [44], antiulcer [45] and antitumoral [46] properties has been reported in the literature. It is obtained from a deacetylation reaction of chitin. Some of the strengths of the chitosan are that, it can assume numerous conformations and it can be attached with a broad range of functional groups to meet specific applications [47]. Its cationic nature, becomes the reason of its significance from the biomedical application perspective [48]. Although, obtaining defect-free chitosan nanofibers still represents a major hurdle, this issue has been tackled by the use of several cosolvents and copolymers [49,50]. The presence of amine group in molecule makes it a weak base which adds another limitation of its insolubility at higher pH. Native chitosan also has relatively poor transfection efficiency and lack of some functionalities which are highly desirable for few TE applications. Therefore several chemical alteration techniques have been tried in order to subdue these weaknesses of chitosan. Graft copolymerization is most frequently used technique among others for chitosan [48].

Wang et al. fabricated composite nanofibrous membrane of chitosan and polyvinyl alcohol using electrospinning for application in wound healing. Antibiotic was loaded in nanofibers at different concentrations. These nanofibers were found to have more and larger nanobeads with increasing concentration of chitosan. The nanofibrous composite was observed to be promising candidate for skin tissue regeneration [50].

#### 3.1.5. Hyaluronic Acid

Hyaluronic acid (HA) is a kind of non-protein glycosaminoglycan which is a large water loving, biodegradable and biocompatible molecule. Characters which make it peculiar biopolymer for application in TERM are its unique viscoelastic properties [51]. HA is another main component of ECM besides collagen [52]. Nevertheless, high viscosity and interfacial tension of HA aqueous solutions, even at low concentrations, make electrospinning a challenging task [53]. Furthermore, insufficient drying of nanofibers during electrospinning due to the robust water holding capability of HA may cause troublesome fusing of electrospun nanofibers on the collector [54]. Therefore, the exploration of a solvent system which will facilitate the electrospinning of HA nanofibers is essential.

Hussein et al. fabricated L-arginine loaded polyvinyl alcohol—HAelectrospun nanofibers for wound healing purpose. Polyvinyl alcohol was blended with HA to promote its electrospunability and citric acid was used as cross-linking agent to improve nanofibers’ resistance against degradation in aqueous environment. Poor mechanical properties of the nanofibers were found to be significantly improved by incorporating cellulose nanocrystals as nanofiller. Developed nanofibers exhibited excellent heamocompatibilityand prominent wound healing effect [23].

#### 3.1.6. Silk Fibroin

Silk fibroin (SF) is a unique natural protein obtained from silkworm silk. Considering many desirable physiochemical characteristics of SF i.e., excellent biocompatibility, biodegradability, resorbability, low immunogenicity, and tunable mechanical characteristics, it has been explored as a potential biopolymer for TE [55]. Silk fibroin carries the property of tailorable degradation rates providing the functional life to scaffold from hours to years. It also exhibits noteworthy mechanical properties when fabricated into different forms [56]. Manufactured scaffolds possess resistance against tensile and compressive forces and have mechanical performance analogues to biological tissues. Their outstanding mechanical properties includes high elongation at break, great strength and toughness [57]. SF has been suggested as one of the best biomaterials for skeletal tissue regeneration [58]. It also shows desirable permeation ability for the exchange of nutrients and wastes [59].

Electrospun SF/kappa-carrageenan nanofibrous membranes were developed byRoshanfaret al. for bone regeneration purpose.Genipin was used as crosslinker which facilitated more crystalline and stable structure of SF. Blending of kappa-carrageenan in nanofibersefficiently moderated the hydrophobic nature of SF-based nanofibers, thus enhancing cell survival and proliferation. The scaffold was able to guide the differentiation towards osteogenic lineage, stimulate the mineralization and development of bone tissue in vitro [24].

### 3.2. Synthetic Polymers

Numerous polymers of synthetic origin have also been tested for the fabrication of nanofibers. The excellence of synthetic polymers which explains its use alone or in combination with natural polymer is due to their features such as its fitness to spinning, excellent mechanical strength and cost-efficiency [60]. Synthetic polymers that are broadly investigated in the fabrication of nanofibers for application in TERM are polycaprolactone (PCL), polyvinyl alcohol (PVA), polyethylene oxide (PEO), polylactic acid (PLA), polyglycolic acid (PGA), polyglycerol sebacate and polyurethanes. The characteristics of the individual polymers are decided by their respective composition and molecular architecture including arrangement of side chains. Biodegradability of the polymers is directed by the characteristics such as chain length, degree of branching and crystallinity [61]. 

#### 3.2.1. Polycaprolactone

PCL is a highly endorsed synthetic biopolymer owing to its FDA approval. It is frequently studied biodegradable polymer which possesses properties such as adequate mechanical strength and tailorable hydrophobicity. Blends, copolymers and composites of PCL with other polymers can be manufactured to achieve desirable physiochemical and mechanical properties [61]. Hydrophobic nature of PCL reduces cell affinity towards PCL surface. Thus, lack of cell-scaffold interactions causes inadequate cells attachment, migration, growth and differentiation, and conclusively results into very slow tissue regeneration [62]. But interfacial characteristics of PCL nanofibers can be altered for TERM usability by making desirable surface alterations in addition to mixing with other polymers [63].

A study aimed at developing dermal equivalent scaffold, fabricated PCL electrospun nanofiber and assembled it with polyethylene glycol diacrylate, sodium alginate and type I collagen (CG1) to fabricate three kinds of dermal equivalent scaffolds. These three group of nanofiber matrices were analyzed for cell viability, adhesion and differentiation and rheological properties, which revealed that the combination of CG1 and PCL is the best suited as dermal equivalent and has potential to be used as graft for dermal regeneration [25].

#### 3.2.2. Polylactic Acid

Wide application of PLA in TE is not only due to its peculiar cytocompatibility and biodegradability [64], but also by the virtue of its chirality. Enantiomers of lactic acid i.e., L- and D-lactic acid can be synthesized having different stereoregularities, which in turn governs the physical and chemical attributes of the polymers, like thermal and mechanical features as well as degradation aspects [65]. Biologically inert and hydrophobic nature of PLA leads to low cell adhesion and lower rate of degradation. Another drawback linked with the usage of PLA is acidic degradation products that causes inflammation at the site of implant [66] These shortcomings hinder PLA’s application in tissue-regenerative treatments. Further research is needed to overcome mentioned drawbacks.

A terpolymer having aniline, dopamine and lactide was used to create conductive nanofibrous scaffold for bone tissue engineering. Adequate physicochemical characteristics such as mechanical, conductivity, electroactivity, wettability, and morphology, along withgood biological properties, made the nanofibers made from this terpolymer a budding candidate to manufacture scaffolds for TE applications [26].

#### 3.2.3. Polyglycolic Acid

Apart from biocompatibility and biodegradability, PGA posses features such as predictable bioabsorption and hydrophilic nature [67]. For the electrospun PGA nanofibers, it has been observed that large surface area of nanofibers brings about speedy degradation and faster loss of strength [68]. Thus PGA happens to be wise choice when a scaffold is expected to be tough initially possessing high strength and elasticity but degrades at a faster rate for quick resorption. However, accompanying sharp increase in localized pH caused by high rate of degradation may induce unwanted tissue responses. Such undesirable tissue responses may precipitate if the region is lacking sufficient buffering capacity or enough means for the rapid elimination of metabolites [69]. Absence of a methyl group in molecular structure of PGA compared with the molecular structure of PLA, makes it more hydrophilic and demonstrate lower solubility in organic solvent. In case of PLA, presence of methyl group creates steric hindrance making it less labile to hydrolysis. Absence of such steric hindrance for PGA leads to faster degradation rate [70]. PGA and PLA are stiff materials which render them unsuitable polymers to fabricate matrices for engineering of soft tissues [68]. PGA is commonly copolymerized with PLA to form poly(lactic-co-glycolic acid) (PLGA.) PLGA is one of the extensively utilized biodegradable polymer on account of its adjustable mechanical characteristics and rate of degradation by varying the lactic acid to glycolic acid copolymer ratio [71]. A terpolymer of lactide, glycolide and caprolactone has been utilized to manufacture porous scaffold for TE purpose. This terpolymer has shown to maintain their dimensions, porous microstructure and mechanical strength for 6 weeks in phosphate buffered saline, even after topographical changes at the surface [72], but further exploration of this terpolymer is needed for application TE.

#### 3.2.4. Polyvinyl Alcohol

PVA has been utilized for TE scaffold fabrication owing to its chemo-thermal stability, mechanical efficiency and its aqueous solubility along with excellent biocompatibility and biodegradability [73]. PVA based scaffolds are noted for maintaining mechanical integrity with ability to withstand high tensile stress, exhibiting good percent elongation as well as high flexibility [74]. PVA is obtained from the hydrolysis or alcoholysis of polyvinyl acetate, thus different grades with varied degrees of hydrolysis are available. PVA grades obtained from high degrees of hydrolysis demonstrate low solubility in water, thus offers high water resistance. One of the limitation of the PVA is its’ hydrophilicity, and thus its’ immediate dissolution on contact with water. This limitation necessitates modification of PVA fibers by chemical or physical crosslinking to enhance its mechanical performance and resistance to water [75]. Another limitation of PVA is poor cell adhesion owing to its low affinity to protein [76], which can be improved using techniques such as blending with macromolecules like chitosan, fibronectin, etc and surface chemical modification such as amination [77].

Asiriet al. fabricated multilayered PVA electrospun nanofibers with epidermal growth factor and fibroblast growth factor to act as biological wound dressing scaffolds. Incorporation of growth factors improved the wettabilty of the PVA nanofibers and stimulated cell adherence and proliferation. This multilayered scaffold showed wound reduction in one week and wound repair in 2–3 weeks, thus exhibiting the potential to be used as biological dressing scaffold [28].

#### 3.2.5. Polyphosphazene

Polyphosphazene symbolizes next generation of biocompatible and biodegradable biomaterials as the excellent design pliability of polyphosphazenes enables the designing of tunable polymers. In addition, such polymers allows to be employed solely or as composites with other polymers to accommodate needs of the application [78]. Chemical groups added to the polyphosphazene backbone chiefly controls the physico-chemical attributes of the polymer [79]. Thus the degradation rate and mechanical stability are controllable with alterations in side groups attached to core molecule. In addition to being biodegradable, the polyphosphazene polymer degrades into products which are non-toxic. Moreover, degradation products do not alter the pH of surrounding tissue because of the buffering capacity of phosphates and ammonia produced during polyphosphazene degradation [80]. Its buffering ability have also been used for neutralization of the acidic byproducts originated from degradation of polymers such as PLGA [81]. Polyphosphazene-polyester blends are drawing attention for TE applications due to non-toxic and neutral pH degradation products along with their controllable degradation pattern [82]. In polyphosphazene, the main chains are flexible due to alternating nitrogen and phosphorus atoms, but it also causes the fiber to shrivel during electrospinning. Surmounting this drawback to get a mechanically sound fiber is a challenge. Many studies which tried to solve this problem aimed at altering molecular structure by addition of large side-groups [83], while others experimented with blend of polyphosphazene with more rigid polymers [82].

Deng et al. fabricated electrospun fibers from dipeptide polyphosphazene-polyetser blend to mimic collagen fibrils. 3D scaffold was designed with concentric alignment of nanofibers with an empty central lumen. These blend nanofibrous scaffolds were shown to support osteoblast adhesion and proliferation and demonstrated an enhanced phenotype expression compared to nanofibers fabricated out of polyester alone. The 3D structure also encourages ECM secretion, indicating its potential for bone regeneration [29].

## 4. Biofunctionalization of Polymers

As discussed in previous paragraphs, most of the natural polymers used to construct scaffolds retain some form of similarity with the ECM found in tissues, but these polymers lack the required attributes such as mechanical strength, adequate stability in vivo and elasticity for its application in TE. Thus, investigators have incorporated synthetic polymers for their favorable mechanical qualities such as strength and elasticity, along with other desirable features of hydrophobicity and slow degradation rate. But synthetic polymers are also ridden with many drawbacks such as inadequate cellular interaction and nonresponse toward tissue integration. These challenges linked with synthetic polymers are due to the structural differences at molecular level which leads to lack of cell surface recognition sites.

One of the way to get around this barrier is surface alteration with biomolecules, where the bulk properties of the polymer especially elasticity and its ability to withstand stress remain unaffected, although alterations in the surface confer necessary characteristics. Such superficial modifications favor an enhanced cellular adherence, causing a drastic improvement in cellular proliferation and supports faster integration of the implant in vivo [84].

Surface modification using biomolecules has remained one of the preferred methods for the advantages it provides in tissue regeneration. Such biofunctionalization involves immobilization of biomolecules on the polymer matrix surfaces to promote cell adhesion and proliferation. Preferred biosignal molecules used for immobilization are cell-growth-factor proteins, therapeutic proteins and cell-adhesion-factor protein [85,86]. Such biomolecules for immobilization includes growth factors, peptide sequences (RGD), natural ECM proteins (fibronectin, laminin, collagen), heparin, heparin sulfate binding peptides among others [87]. Besides providing structural backbone, the scaffolds modified with ECM components initiate cellular interactions which are decisive for cell attachment, growth and differentiation [88].

Numerous techniques have been worked out for physical or chemical immobilization of such protein molecules. These are grafting, polymer blending and chemically modifying the polymers. To comprehend about the biofunctionalization of polymers, is it necessary to be aware about the composition of the ECM.

ECM is a complex network comprised of a cluster of macromolecules organized according to tissue type. It is composed of two prime families of macromolecules: fibrous proteins and proteoglycans (PGs) [89]. Collagens, elastins, fibronectins and laminins are the fundamental fibrous ECM proteins [90]. Collagen is the principal structural element of the ECM and the most extensive fibrous protein forming the ECM. It makes up about 30% of the total protein weight in animals and perform an array of functions such as providing resistance to breaking under tension, controlling cell adhesion, assisting chemotaxis and directing development of tissues [91]. Collagen is accompanied by elastin, which is another essential ECM fibrous protein. Elastin confers recoiling property to those tissues which undergoes frequent stretching. Fibronectins are engaged in guiding the arrangement of ECM with an essential role in facilitating cell attachment. These proteins are associated together by proteoglycans and makes up the thin fibers of the ECM [92]. Proteoglycans (PGs) are constituted of glycosaminoglycan (GAG) chains linked to a core protein with covalent bonding. Proteoglycans perform an important function of signal transduction by binding various signal molecules and regulate many cellular processes, in addition to being a structural protein [93]. GAGs are highly water loving and adopt immensely extended conformations that lead to development of hydrogels. The matrices formed by GAGs are capable to withstand high compressive forces [90].

Modification of the polymers with ECM proteins and growth factors is a commonly followed strategy. Table 3 summarizes biofunctionalization with a range of bioactive molecules, methods used for biofunctionalization and outcome of biofunctionalization. Forthcoming paragraphs will review biofunctionalization with various molecules and the improvements achieved using such biofunctionalization.

Collagen (type I) is the most copious extracellular protein and it exists in a nanorange fibrillar structure. Such fibrillar morphology has been demonstrated to be crucial for attachment of cells, their growth and differentiation [116]. Collagen is one of the most favored bioactive molecules used for coating, as it provides the biomimetic environment for cell life cycle. Duan et al. constructed PCL nanofibers using electrospinning and layered it with collagen to merge the desirable attributes of collagen and PCL. PCL possess superior mechanical characteristics, yet its hydrophobicity and poor cell affinity results into poor cell attachment and proliferation. Collagen was immobilized on PCL nanofibers with the aim to improve the cell affinity of nanofibers after surface modification using remote plasma treatment. This study indicated that collagen immobilization along with plasma treatment offered an significant enhancement in surface hydrophilicity and greatly improved the primary human dermal fibroblasts (HDF) attachment and growth compared with pristine material [94]. In another study, collagen coated PCL nanofibers were prepared using two different methods, using coaxial electrospinning technique to give core-shell structure and by soaking the PCL matrix in collagen solution to form a rough collagen coating over PCL nanofibers. Although both kind of collagen immobilization over PCL nanofibers favored cell proliferation, HDF density found more over the nanofibers with core-shell structure compared to simple collagen coating over nanofibers [95].

The inclusion of Gelatin in scaffolds enhances the characteristics such as cell attachment, cell growth and biomineralization. Coating of the polymer matrices using gelatin resulted into enhanced biocompatibility and mechanical performance [117]. Such coating with gelatin also suppresses the activation of the complement system and opsonization, thus reduces immunogenicity of other polymers in matrix [118]. The presence of gelatin improved cellular proliferation of mouse embryonic fibroblasts (MEF) in electrospun PCL nanofibers blended with gelatin and those coated with gelatin, but the highest improvement was observed for nanofibrous scaffolds prepared using blend of PCL and gelatin [119]. Safaeijavan et al. altered the surface of PCL nanofibers by gelatin grafting to enhance their compatibility with living medium. For grafting, PCL scaffolds were initially given air plasma treatment which adds carboxyl groups on polymer surface. Gelatin molecules were then covalently grafted on nanofiber, which inserted amine functional groups on the surface. Such grafting not only increased the hydrophilicity of the scaffold but also enabled the scaffold to hold fibroblast cells and support their survival and functioning [96].

Fibronectin is large adhesive glycoprotein of the ECM essential for cell functions such as adhesion, spreading and motility. In a study, the functionalization of PCL electrospun fibers with fibronectin was achieved using three different approaches—protein surface entrapment, chemical functionalization and coaxial electrospinning. Improved cell adhesion and proliferation of bone murine stromal cells was obtained for scaffolds functionalized using all the three approaches. But sample with the surface entrapment of fibronectin demonstrated better performance in terms of cell response, which indicated that surface entrapment was the best approach to attain efficient functionalization for electrospun fibers [97]. Xie et al. fabricated scaffolds of PCL nanofibers with radial alignment. Influence of fiber alignment and fibronectin surface layering on cell motility of fibroblasts was studied. It indicated that fibronectin coating was able to boost the effect of topographic cues offered by the fiber alignment on cell morphology. Even in the case of randomly aligned nanofibers coated withfibronectin, cell adherence and distribution were enhancedcompared to the unlayered sample [98].

One of the most frequently employed peptides is RGD (arginine-glycine-aspartic acid) which originates from fibronectin. RGD is the leading integrin-binding domain situated inside various ECM proteins such as fibrinogen, fibronectin, vitronectin, osteopontin, bone sialoprotein as well as in some laminins and collagens [120]. It not only regulates the endothelial cells adhesion, migration and proliferation but also can be utilized to preferentially focus on certain cell lines and bring out specific cell responses. The grafting of short peptide sequences like RGD has some benefits when compared to entire protein molecules, such as greater stability under sterilization processes, storage, heat application, pH alterations and against enzymatic degradation. Short peptides also has lower space requirement, which leads to a higher density packaging of the peptides [121]. But RGD is recognized by numerous integrins, thus acts as a nonspecific peptide [122]. Choi et al. developed electrospun nanofibrous matrix of polyurethane over which RGD peptides were immobilized to enhance affinity of endothelial cells. RGD-immobilized matrix exhibited improved viability of human umbilical vein endothelial cells in comparison with an unaltered surface, proving that immobilization of RGD peptide has benefitted cell proliferation [99]. Besides RGD, several other cell adhesion motifs have been recognized namely DGEA peptide from collagen, GREDVY, KQAGDV peptide from fibronectin, PHSRN, etc. [121]. Thus, the RGD sequence can not be considered as the “universal cell recognition motif”, nevertheless it is one-of-a-kind given its broad distribution and usage.

Laminin (LM) is heterotrimeric glycoprotein having high molecular weight. It is an essential constituent of basement membrane lining many tissues. This glycoprotein is necessary for activities like cell attachment, survival, growth, mobility and specialization [123]. Junka et al. developed electrospun nanofibers for tissue regeneration in large-gap peripheral nerve injury. Nanofibrous scaffolds employed blends of PCL and chitosan. Functionalization of the scaffold surface with laminin was done by crosslinking and by using conventional adsorption method. Schwann cell attachment and proliferation rates were found to be significantly greater on laminin crosslinked to PCL-chitosan scaffolds in comparison to scaffolds adsorbed with laminin or scaffolds without laminin [105]. Incorporation of Laminin in scaffolds has been tried for the regeneration of many diverse tissues including intervertebral fibrocartilage, muscles, neurons and blood vessels [123].

The natural adhesion between the ECM and cells generally depends on the creation of integrin-interceded bonds between integrins in the cell membrane and adhesion proteins or motifs in ECM. Here, the presence of cell membrane integrin controls the efficiency of cell adhesion. However, avidin-biotin linkage is an extrinsic, integrin-independent, high affinity receptor-ligand complex. This avidin-biotin system can be utilized for improved seeding of the cells into scaffolds. Given approach is founded on the existence of multiple binding sites on avidin for biotin and the strong non-covalent interaction between them. In TERM applications, biopolymer matrices are conjugated with avidin and cell membranes are attached with biotin to enhance cell interaction with matrices. Pan et.al evaluated avidin-biotin technology with poly(caprolactone-co-lactide)/Pluronic (PLCL/Pluronic) nanofiber based scaffolds for improving cell adhesion. Nanofiber surface is coated with avidin, whereas cellular membrane is attached with biotin. This research showed the improved adhesion and proliferation of stem cells on nanofiber matrix with the aid of technique based on avidin-biotin complex [106].

After in vivo exposure of scaffold, fibronectin and vitronectin gets adsorbed on the surface of scaffold non-specifically. By virtue of such adhered ECM proteins, the cell-scaffold interaction improves. Such interactions are controlled by integrins, which are cell surface receptors principally involved in attachment of cells to ECM [86].

Another commonly exercised approach in TE to bring out cellular differentiation is the utilization of growth factors. Yet, the constraints linked with the use of growth factors, such as rapid blood clearance, large dose requirement and heavy price, have aroused the exploration of growth factor substitutes, including mimicking molecules. Insulin has been examined as a biochemical signal due to its structural alikeness with Insulin Growth Factor-1 (IGF-1) and similarity between their receptors [124]. Ramos et al. developed insulin functionalized scaffolds where insulin was immobilized on polycaprolactone—cellulose acetate electrospun fiber matrices. The cells incubated on insulin conjugated scaffolds presented a rise in tendon markers, indicating potential of its use for tendon repair and regeneration [108]. In another study, a significantly increase in collagen I and III was observed postsurgery where bioactive insulin-immobilized electrospun nanofiber matrices cultured with mesenchymal stem cells were sutured to transected Achilles tendons in animal model. Furthermore, these matrices promoted alignment of collagen fibrils in regenerated tendons [125].

Mussle inspired peptides have attracted significant attention to functionalize material surfaces because it caters a simple and flexible approach and eliminate the requirement of expensive or complex instruments and procedures. Mussle inspired chemistry is founded on catechol-effectuated molecular adhesion [126]. Polydopamine (PDA) is one of the mussle inspired molecule. Chen et al. successfully used PDA to mediate bromelain immobilization on electrospun PCL fibers. Purpose of such immobilization was to apply antibacterial, anti-inflammatory, anti-edematous activities of bromelein and its capability to hydrolyze necrotic tissues to augment rates of wound healing. Bromelain–polydopamine–polycaprolactone (BrPDA-PCL) fibers exhibited superior biocompatibility given the hydrophilicity of the PDA coating which provides a suitable surface for cell adhesion. BrPDA-PCL fibrous membrane was observed to be highly effective wound dressing. It exhibited antibacterial activity, in addition to assist both cellular adhesion and proliferation [110]. In another study, mussel-inspired polynorepinephrine (pNE) was used to coat PCL fibers to improve hydrophilic nature and cellular interaction of hydrophobic surfaces. pNE functionalization created suitable environment both in vitro and in vivo for skeletal muscle cell adhesion and proliferation [111]. pNE coating has been also been utilized to create bio-interface by applying smooth coating of pNE on electrospun Poly(lactic acid-co-caprolactone) fibers. Here, the catechol groups from pNE assisted in collagen anchoring to improve cell adhesion and to immobilize nerve growth factor to advance differentiation to neurons [112].Polyphenol is another biomolecule whose addition in nanofibrous scaffolds increases cell adhesion, proliferation and differentiation, along with exhibiting their antioxidant and antimicrobial activity. Many polyphenols such as curcumin, naringin, apigenin, icarrin have been studied for bone tissue regeneration, which indicates their prospective for use in TE [127].

Along with the improvement in cell adhesion and proliferation with adoption of biofunctionalization using different approaches as seen in earlier paragraphs, further improvement in tissue regeneration can be achieved with the use of various growth factors. The simultaneous deliverance of angiogenesis-related factors and other biomolecules by nanofibrous matrices has demonstrated to boost tissue repair and regeneration [128]. Angiogenesis is of a pivotal occurrence in tissue regeneration which is essential to carry out the functions such as delivery of oxygen, nutrients, growth factors, ligands and disposal of metabolic byproducts. Therefore, numerous bioactive molecules have been incorporated in biomaterials to impart angiogenic activity. Scaffold-based transfer of vascular endothelial growth factor (VEGF) and bone morphogenetic protein 2 (BMP2) is the commonly investigated combination to promote angiogenesis and osteogenesis owing to their respective pro-angiogenic and osteoinductive activities [129,130]. Kai et al. fabricated PCL-gelatin (PG) nanofibers in which VEGF was incorporated using two individual methods namely blending and co-axial electrospinning to induce the cardiac differentiation of cells. The VEGF incorporated nanofibers improved the cell growth and division of mesenchymal stem cells (MSCs), promoted cardiac differentiation of MSCs and helped in enhancing the translation of cardiac-specific proteins [114].

VEGF has reported to be angiogenic and promoted formation of natural bypasses in cases of myocardium infarction by promoting generation of neovasculature and dissolution of existing vasculature [131]. Many recent findings in TERM offer proof that surface immobilization of growth factors helps in induction of activity for prolonged duration. Guex et al. used electrospinning for fabricating PCL nanofibrous constructs and VEGF was covalently bound to it. On evaluation of its effect on cell division of endothelial cells in vitro, it was observed that number of endothelial cells were noticeably increased on VEGF-immobilized scaffolds in comparison to non-functionalized PCL scaffolds, suggesting that biological activity of immobilized VEGF was maintained [115].

Epidermal growth factor (EGF) induces growth, proliferation, differentiation as well as cell survival by binding with its membrane receptor [132] and is considered the frontrunner in advancement of wound healing [133]. EGF facilitate wound healing by improving epidermal and mesenchymal restoration, cell migration, proliferation and ECM regeneration [133]. PVA electrospun nanofibers were fabricated to act as biological wound dressing scaffolds by Asiri et al. EGF and fibroblast growth factor (FGF) were incorporated in the PVA nanofibers which resulted in the improvement in wettability and surface roughness. Growth factor release from the PVA nanofibers resulted in stimulation of cell adhesion, proliferation and improvement in cell viability. In vivo evaluation showed that GFs added PVA nanofibers expedited the healing process in burn wound by boosting epithelialization and proliferation of dermal fibroblasts [28].

## 5. Fabrication Techniques of Nanofibers

Diverse ways has been explored to fabricate nanofibers, some of which are template synthesis, phase separation, self assembly, interfacial polymerization and electrospinning [134]. Apart from the selection of the material from the broad range of polymers for fabricating nanofibers, the management of nanofiber diameter is extremely decisive in biomedical applications, as it decides the surface area for cellular interactions. Alongside fiber diameter, other attributes namely fiber morphology, architecture and alignment are also the significant variables instrumental in deciding the cell-fiber interactions for biomedical applications [16,135]. Above mentioned are some of the parameters used in selection of nanofiber fabrication technique. Scalability to the commercial scale is another crucial factor to consider while selecting fabrication technique. Among the mentioned techniques, electrospinning is the extensively experimented nanofiber fabrication technique and it has offered the most encouraging outcomes for TERM applications. Nanofiber synthesis using other techniques for TE application has been studies on relatively limited basis.

In Phase separation technique, nano-fibrous matrices are prepared following a process that involves polymer dissolution, thermally induced gelation, exchange of solvent, freezing and freeze-drying [136]. Gelation is the decisive stage in this technique for the creation of fibrillar matrix. Gelation of polymer solution depends upon solvents used, polymer concentration and gelation temperature. Gelling temperature is another critical element influencing the porous structure of the fibrous matrices. Porosity up to 98.5% had been achieved using this technique [137]. Some of the strengths of phase separation technique are minimum requirement of sophisticated equipments, simplified procedure, ability of the method to produce fibers in nanorange and capability to construct fibrous scaffold matrix in the anatomical shape of the body part using mold.

Biomolecular self-assembly presents an easy way to manufacture functional nanomaterials. The self-assembly mechanisms of biomolecules are based on varied internal interactions, such as hydrogen bonding, electrostatic interactions, hydrophobic interactions, π–π stacking, ligand–receptor binding and DNA base pairing. In addition, self-assembly can be induced using external stimulations like making alterations in solution attributes such as pH, ion concentration and temperature, by addition of organic solvents or enzymes, and with the help of light [138]. Self assembly is a bottom up technique to manufacture nanofibers in which molecules tend to align themselves in specific patterns to generate nanofibers. The structure of the individual molecules taking part in self-assembly and the intermolecular forces involved in molecular interactions decides the morphology of the nanofibers. This technique is capable of producing fibres in nano range. Yet the drawbacks such as low productivity, arduous handling of the fiber dimensions, constricted choices of materials which can self-assemble and being a cumbersome process, this method is of least preference [134].

Template synthesis of nanofibers involves extrusion of polymer precursor solution from the nanoporous membrane into the solidifying solution under pressure. As soon as polymer solution touches solidifying solution, nanofibers are created. Nanofibrous membrane containing cylindrical pores is used as as a template/mold. Aligned nanofibers with different diameters can be fabricated using templates with different pore diameters [139]. Limitation of this technique is the formation of discontinuous fibers having variable diameters.

Interfacial polymerization is another method of generating nanofibers, which is mainly a polycondensation reaction happening at the interface between two kinds of monomers solubilised in two non-miscible solvents. On mixing two distinct phases containing monomers, polymerization happens at the interface of the dispersed phase and dispersion medium of emulsion. Homogeneous nucleated growth is the key determinant in this technique.

### 5.1. Electrospinning Method

Electrospinning has come out as one of the most rewarding techniques, considering its capability to manufacture fibers in the nanometer range, having morphology which is comparable to the ECM fibrous structure. It also provides control over the fiber diameter, its composition and the porosity of nanofiber meshes [136]. Furthermore, this technique is remarkably simple, robust, and versatile, thus making it the preferred choice for preparing nanofibers. Polymer can be selected from a wide range of materials amenable to electrospinning and this technique has been employed by now to manufacture nanofibers of more than 100 different kinds of polymers [140].This method is adept for scaling up to make production at commercial scale [141]. Since the attributes of electrospun nanofibers are highly manageable and can be customized to befit various tissues or to encapsulate different drugs, such fabricated nanofibers are highly resilient for use in different biomedical applications.

**Figure 2:**
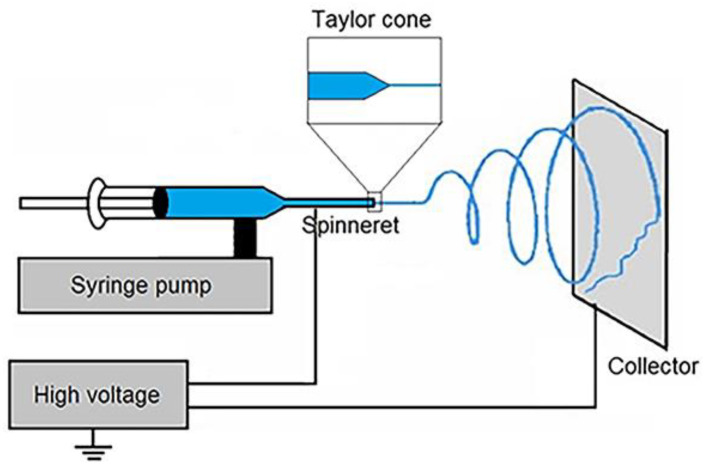
Schematic representation of typical electrospinning system [144] ©2016 Yawen Li and Therese Bou-Akl. Originally published in “Chapter 6 Electrospinning in Tissue Engineering”from Book “Electrospinning—Material, Techniques, and Biomedical Applications” under Creative Commons Attribution License (http://creativecommons.org/licenses/by/3.0, accessed on 4 December 2022). Available from: https://doi.org/10.5772/65836, accessed on 4 December 2022.

The electrostatic repulsion between polymer molecules help in overcoming surface tension of the polymer solution, drawing a jet from polymer solution drop and further stretching of the jet is the principle on which electrospinning is based. Such electrostatic repulsion between polymer molecules develops with the help of electrical potential difference applied between the electrodes. A symbolic electrospinning setup as shown in Figure 2 comprised of following essential components: a higher voltage potential current, a syringe with needle tip, a pump and a metal collector [142]. On the induction of voltage between the needle and the collector, charges begin to develop on the polymer molecules in the needle. The magnitude of charge in the polymer molecule determines the extent of electrostatic repulsion experienced by the molecules of polymer in polymer solution. Surface area of the polymer solution increases as the electrostatic repulsion between polymer molecules increase [16]. But to produce fibers in nanometer range, charges on the polymer molecules should be dense enough and at the same time it should not that high to cause the solution jet to split into droplets [143]. The electrostatic repulsion developed between polymer molecules opposes the cohesive forces between polymer molecules and led to the formation of Taylor cone. This Taylor cone then turns into charged jet, which stretches, thins out and finally collects on the metal surface. All along the travel of polymer solution from Taylor cone to the collector, solvent evaporation provides rigidity to the fiber. The solvent evaporation mechanism also influences the porosity of the fibers [16].

The parameters which influences the characteristics of the nanofibers obtained can be broadly divided into parameters related to electrospinning solution, parameters related to processand environmental parameters. Polymer solution concentration controls viscosity, surface tension as well as charge density and is thus the prime parameter deciding fiber diameter [145]. Other solution related parameters includes polymer molecular weight and distribution and solvent or mixture of solvent used. Process parameters include parameters related to equipment set up namely orifice diameter, voltage, solution feed rate, spinning distance, design of collector and motion of collectorthat affects the nanofiber attributes. Apart from these, the ambient factors like temperature and humidity, which are covered under environmental parameters affect the quality of nanofibers obtained. High humidity lengthens the solidification time required by the fibers after their ejection from the needle orifice, whereas low humidity assists in efficient removal of the solvents from the nanofibers. Humidity also influences the surface morphology of the fibers, with increase in humidity has been reported to cause an increase in number and size of the pores in the nanofibers [146,147]. Increase in ambient temperature has been reported to cause reduction in diameter of electrospun fibers [148]. Yet, the influence of the environmental factors on the properties of fibers should be studied on case by case basis.

Fridrikh et al. presented a model that predicts terminal jet diameter based on the availability of information on flow rate, applied voltage, and interfacial tension of the liquid. Fiber formation in electrospinning is a result of counterbalance between polymer adhesive forces due to surface tension and repulsive forces due similar charges on polymer molecules. On this relation, the prediction of fiber diameter has been based [149]. However, electrospinning is also linked with certain shortcomings such as wide range of electrospun fiber diameter, irregular alignment of fibers, and poor mechanical performance of the fiber matrices [135].

**Figure 3:**
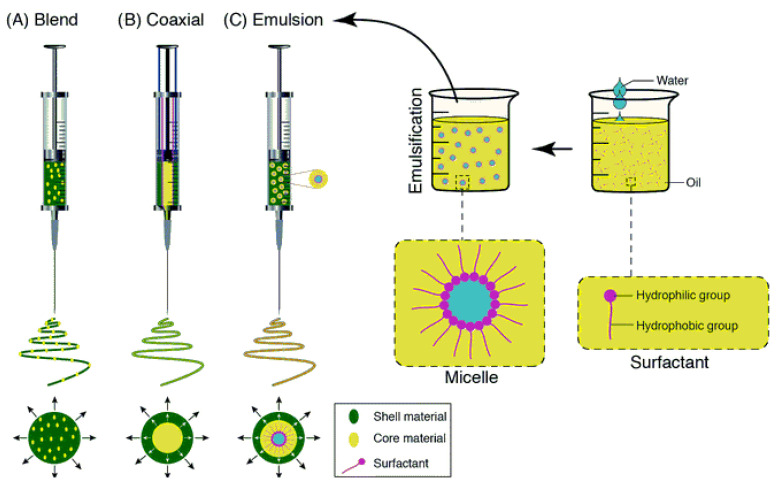
Electrospinning techniques—Blend, Co-axial and emulsion electrospinning. Reproduced from reference [150] with permission from Royal Society of Chemistry.

Electrospinning method can be classified according to the kind of nozzle used into three classes-single nozzle, coaxial and multi-nozzle electrospinning, whereas according to the kind and number of solutions or melts used, it can be categorized into blend electrospinning, co-axial and emulsion electrospinning as shown in Figure 3.

#### 5.1.1. Single Nozzle Electrospinning

Single nozzle electrospinning uses a nozzle with single aperture through which polymer melt or solution outflows. Composite nanofibers can be manufactured using this mode of electrospinning. Compatible polymers can be employed for electrospinning of polymer blends. Moreover, solid nanoparticles can be embedded within electrospun fibers and liquid phase particles can be electrospun using emulsion electrospinning method. Single nozzle electrospinning method uses either blend of bioactive molecules in polymer dissolved in solvent, melt of polymers or emulsions for electrospinning.

#### 5.1.2. Co-Axial Electrospinning

Co-axial electrospinning produces core-sheath fibers by physically separating them using two co-axial electrospinning needles and two solutions. Co-axial electrospinning uses the concurrent flow of different solutions through two co-axial capillaries to physically separate core and shell fibers. Specific processing parameters such as solution flow rates and solution properties like viscosities and electrical conductivities are typically taken into account while attempting to apply the co-axial approach. For example, the compositions of the fiber’s core and sheath may be chosen depending on their ability to provide strength and their ability to support cells respectively. Selection of polymeric material and solvent is of importance for consistent generation of coaxial fibers. Viscoelasticity of the polymer solution forming shell should be sufficient to stabilize the fluid jet to create core-shell morphology of the fibers. A study shows that the morphology of the nanofibers depends on the interaction between the core and shell solutions during co-axial electrospinning, rather than their individual effects. If two highly miscible solutions are used, then partial mixing of those solutions occurs during co-electrospinning, which significantly influences the morphology of resulting nanofibers [151].

This technique can be applied to even non-electrospinnable materials, which can form the core of the core-shell nanofibers, whereas solutions forming the shells should possess spinnability. Moreover, active compounds devoid of fibrous characteristics can also be enclosed in the fiber core. The technique also offers the benefit of building a single drug delivery system from two or more bioactive compounds with varied biological activity and solubilities. Coaxial electrospun fibers with topographical and biochemical features are utilized for TE applications. Drugs are often incorporated in the core and released by shell polymer degradation or sheath pores. A longer-lasting drug release is possible using coaxial electrospinning of drugs and polymers. The sheath barrier effect can stop an initial burst discharge of the drugs [152]. Core–shell nanofibers have also been explored for the dual discharge of growth factors, wherein growth factor incorporated in core followed a time-controlled release compared to the growth factor attached on the shell surface [153]. Dual drug loading has also been achieved using core-shell nanofibers by loading drugs each in the core and the shell. Core exhibited the long-term release, whereas shell showed short-term release of the drugs to improve the tissue regeneration efficiency of the scaffolds fabricated from such fibers [154].

Triaxial electrospun fibers have also been developed with dual drug delivery capability using modified electrospinning technique. In triaxial fibers, interaction between core-intermediate layers and sheath-intermediate layers contributes to the mechanical strength of the fibers [155].Living cells can also be electrospun into the core of the fibers, encapsulated within polymeric shell. But this kind of electrospinning has been discussed in separate section in this review.

Although it requires a complicated setup, coaxial electrospinning offers numerous benefits like one-step technique for encapsulating, ability to make composite nanofibers, and its applicability for a variety of materials. With all of its benefits, coaxial electrospinning has been extensively utilised in the creation of nanofibers for varied applications [156].

#### 5.1.3. Multiple-Jet Electrospinning

The preliminary form of electrospinning uses a single-needle to efflux the polymer solution and to create fibers. Notwithstanding the range of benefits offered by this simple form of electrospinning, the major drawback of the lower production rate with conventional electrospinning restricts the utilization of the process at a commercial scale. Multiple-jet electrospinning technique has been considered to surmount this deficiency of lower rate of production, but creation of multiple jets carries with it the issues including repulsion between jets, non-uniformity of electrical fields, poor control over the process and decline in fiber quality [157]. This necessitates even further development and optimization of the process. The operating principle of multi-nozzle electrospinning is same as that of conventional single-needle electrospinning technique, with the major difference lies in use of multiple nozzles. In a single setup, multiple nozzles are arranged in various configurations to generate multiple jets [158]

#### 5.1.4. Blend Electrospinning

In Blend electrospinning technique, bioactive materials are solubilised or suspended within polymer solution. Physicochemical characteristics of the solution and its interaction with the bioactive materials decides the disposition of bioactive molecules within fibers [150]. Blend electrospinning method is uncomplicated compared to coaxial and emulsion electrospinning, but it also has some drawbacks such as sensitive bioactive agents may get denatured due to presence of the solvents and thus suffer from loss of their bioactivity [159]. Polymers such as poly(ethylene oxide) (PEO) and poly(vinyl alcohol) (PVA) having good water solubility, have been utilized to encapsulate bioactive proteins [160,161]. Surface accumulation of bioactive molecules is commonly observed in nanofibers, because such molecules are charged and they migrate towards the surface of the jet due to repulsion between them, during jet ejection and elongation.

#### 5.1.5. Emulsion Electrospinning

The emulsion electrospinning involves basic set up similar to that of blend electrospinning but comprises spinning of emulsion. This is another unique and simple approach to electrospin core-shell nanofibers using a single nozzle spinneret. In comparison with coaxial electrospinning which employs coaxial needles to manufacture nanofibers with core-shell morphology, emulsion electrospinning uses single nozzle to electrospun nanofibers, therefore making it simple and more conducive for scaling up [21]. Core-shell structure is obtainable in electrospun nanofibers using either water-in-oil (W/O) [21] or oil-in-water (O/W) emulsions [162] to load respectively hydrophilic or hydrophobic compounds into the core of nanofibers [163]. In this method, polymer is solubilised in organic or aqueous solvent to form the dispersion medium whereas bioactive substances are solubilised in organic or aqueous solvent forming dispersed phase. Formulation of emulsion eliminates the requirement for common solvent for polymer and bioactive molecules. Availability of common solvent is considered as a primary necessity of the blend electrospinning technique, which is omitted in emulsion electrospinning. After ejection of jet in emulsion electrospinning, evaporation of the solvent of dispersion medium from ejected jet increases the viscosity of that phase. As a consequence, droplets of the dispersed phase travel to the core of the jet due to viscosity gradient [164]. Mutual dielectrophoresis caused by electric field led to coalescence of the droplets at the centre of the fiber, thus giving fiber a core–shell morphology. Stability of the emulsion is a decisive consideration for emulsion electrospinning, which necessitates addition of emulsifier to prevent emulsion from breaking down. This technique has been developed to incorporate functional elements such as enzymes, bioactive proteins and drugs. This technique is a potential alternative to conventional electrospinning methods because it enables loading of lipophilic drugs using affordable hydrophilic polymers and bypass the requirement of restricted, less safe solvents [150]. Some other crucial determinants of fiber characteristics include the nature of emulsion, strength of applied electric potential difference, conductivity of dispersed phase, interfacial tension exhibited by emulsion, and cooling time among others [165].

#### 5.1.6. Cell-Electrospinning

On account of aforementioned benefits, electrospinning has earned noteworthy attention for applications in biomedical field. Nevertheless, it carries some constraints with it, such as utilization of toxic solvents, low and uncertain cell penetration and non-uniform cell distribution [166]. In addition, to seed cells in scaffold, it needs to be kept in bioreactor for long durations. Even then, there remains the uncertainty about the distribution of the cells within scaffold. To subdue such limitations, a unique approach, termed cell-electrospinning (C-ES), was invented. Cell electrospinning was discovered in around 2005–2006 [167,168]. C-ES originated from the typical electrospinning technique but is capable of embedding living cells inside the fibers. Use of viable cell in the electrospinning process differentiates C-ES it from conventional electrospinning technique. C-ES allows us to construct fully cellularized three-dimensional tissue construct by directly handling the cells. Cell-electrospun fibers demonstrate primacy by directing the cells along the fibers, enabling an effective and quick exchange of nutrients and gases and providing better cell-to-cell interaction compared to cell-embedded bulk 3D construct. But some of its limitations include inadequate mechanical strength, restrictions in development of 3D structures and less control over cell density [166].

In this technique, one of the approach is to encapsulate the biosuspension containing living cells in the core of a fiber, using a coaxial needle, within a shell fabricated out of a biocompatible polymer [168]. Electrical conductivity of biosuspension flowing through the inner needle and polymer solution flowing through the outer needle make an important consideration for the electrospinning. Viscosity, flow rate of both the liquids and the strength of the applied electric field are critical variables to analyze in this technique. Needle with different configuration such as single as well as tri-needle can also be used. The type of needle used decides the core arrangement, which can vary from single to tri-core morphology. Another consideration is that the ground electrode in cell electrospinning is significantly different compared to those used in conventional electrospinning technique. This modification is to avoid dehydration of the encapsulated cells to avert cellular damage or death [167]. Due to the negative effect of the electric field on the viability of the cells in biosuspension, magnitude of the electric field can not be raised above threshold. Dehydration and shear developed during stretching of the fibers are the probable reasons besides the applied electric field for the low viability of the cells during cell electrospinning process.

A study developed active biological microthreads using coaxial electrospinning method. A concentrated living biosuspension was used to form the core and a medical grade poly(dimethylsiloxane) was used to form the shell of the microthreads. Along the length of the microthreads, cell aggregates generated the capsules. Cell viability assay showed that the viability of the cells passed through the electric field to be around 67%, which was not statistically much different from the viability of the control cells. No indications of any harm to encapsulated cells were observed while generating microthreads through cell electrospinning using co-axial needles [168].

Guo et al. developed an electrospinning approach to enclose cellular aggregates into fibrin/polyethylene oxide microfibers. Encapsulated cellular aggregates within fibrinogen microfibers were suspended into a rotating bath containing thrombin to produce fibrin fibrils by thrombin induced polymerization of fibrin. Researcher established that loading cellular aggregates less than 100 µm in size and adjusting process parameters in electrospinning led to improved cell survival [169]. Considering the great interest developed in the area of cell electrospinning owing to the benefits provided, more of the studies are expected in future.

Table 4 discusses the advantages and limitations of different electrospinning methods reviewed in above paragraphs.

## 6. Applications of Polymeric Nanofibers

Use of nanofibers has been evaluated for range of tissues from a cornea [170], myocardial tissue [171] to skeletal muscles [135]. This review summarizes the applications of nanofibers in regeneration of neural, vascular, cartilage, bone and dermal tissues.

### 6.1. Neural Tissue Regeneration

The peripheral nerve injury creates a major problem in their repair and restoration. Autografting, allografting and xenografting offers recourse to overcome this difficulty. However, donor site morbidity, the lack of donors and low proficiency in grafting techniques turn up to be limitations of these alternatives [172]. In contrast, the electrospun nanofibers offer multiple benefits, including controlled alignment which provides spatial assistance for neurite outgrowth, axon lengthening [173] and mechanical cues for differentiation of stem cells [174]. Apart from this, aligned nanofibers were noticed for supporting Schwann cells migration and thus assist in reestablishing a growth cone at the tip [175].

Afrash et al. developed a nerve growth factor (NGF) functionalized aligned nanofibrous scaffold based on polycaprolactone/chitosan (PCL/CS) polymers for tissue regeneration of neural cells. NGF was used as a neurotrophin and it was attached to PCL/CS nanofibers with the use of dopamine coating. Polydopamine coating reduced the hydrophilicity of the nanofibers, whereas immobilization of NGF on the nanofibers improved the hydrophilic nature. It was observed that, aligned fibers were more hydrophilic compared to randomly aligned fibers. It established that topography and morphology can control interfacial tension. It also demonstrated that regular alignment of PCL/CS nanofibers could provide desirable conditions for neural cell growth [9]. In another study by Xieet al., the characterization of embryonic stem (ES) cell culture on electrospun PCL nanofibers with regular and irregular alignment, manifested the significance of material topography in cell differentiation. PCL nanofibers seeded with ES cells showed that stem cells specialized to oligodendrocytes and astrocytes along with many other neural lineage cells. In addition, this study demonstrated that regular alignment of nanofibers could retard the specialization and maturation of ES cells into astrocytes, which play a critical role inthe repair of spinal cord traumas [176].

### 6.2. Vascular Tissue Regeneration

An impediment of in vitro fabrication of vascular tissues to fulfill the clinical necessity of tissue grafts is lingering since the dawn of TERM [177].The 1950s saw the development of synthetic tissue-engineered vascular grafts (TEVGs) to restore blocked arteries after surgical complications. TEVGs were used as a remedy to the regular scarcity of allogenic tissue grafting and to mitigate the problem of immunological rejections after transplantation. But these synthetic TEVGs were found to be unable to noticeably reduce overall mortality and morbidity [178]. To solve this issue, the researchers have employed various in vitro strategies to prepare vascular tissue having ability to interact with cells to develop new blood vessels [60].

Shin et al. fabricated PLGA nanofibers with co-functionalization of RGD peptide and graphene oxide (GO) for vascular TE using the electrospinning technique. Surface functionalization with RGD and GO on PLGA nanofiber improved hydrophilicity and facilitated interaction between nanofiber and cells. RGD peptide functionalization greatly increased initial attachment and growth of vascular smooth muscle cells (VSMCs). In addition, GO also supported enhanced proliferation of VSMCs. The study shows the promising potential of RGD-GO-PLGA nanofiber matrices for vascular tissue regeneration [179]. Marelli et al.electrospun SF into fibers with tubular morphology for small diameter vessel grafting. These electrospun tubes were able to resist pressure up to 575 ± 17 mm Hg, which is more than fourfold of normal systolic pressure i.e., 120 mm Hg and more than twice that of pathological upper pressure of 220 mm Hg. SF tubes displayed good cytological compatibility in in vitro analysis. Thus, electrospun tubes designed in this study show bright prospects for small diameter blood vessel grafting [180].

### 6.3. Cartilage Tissue Regeneration

Articular cartilage is a functional connective tissue which covers the ends of bones at the site of junction of two or more moving bones. The ECM in cartilage tissue is dense, while chondrocytes are thinly distributed within matrix. Such entrapment of chondrocytes within dense microenvironment prevents its mobility to adjoining regions within cartilage. Though chondrocytes responds to variety of stimuli, it rarely form cell –to-cell contacts for direct cell transduction. Moreover, limited ability of chondrocytes to replicate is responsible for poor regenerative capability of cartilage in case of an injury [181]. In addition, articular cartilage lacks blood vessels, lymphatics and nerves, limiting its ability to regenerate tissue after injury. Damage in the cartilages necessitates replacement most of the times. To settle this problem, researchers have experimented with many TE strategies, including sponges, hydrogel scaffolds, gelatin microsphere, and collagen sponges. These approaches showed limited improvement in the cartilage healing process. Conversely, the nanofibers synthesized from synthetic, natural, and composite polymers provide good results due to its resemblance with the ECM. Such Nanofibers promote the cell-ECM interaction and chondrogenic differentiation. Very high surface area compared to total volume of the aligned nanofibers manifests the potential of engineering articular cartilage using approaches of TE [60,135].

Semitela et al. synthesized the bioactive polycaprolactone-gelatin nanofibers scaffolds (PCL + GEL) with enhanced pore size and interconnectivity for cartilage tissue repair. Polyethylene glycol (PEG) was incorporated during the electrospinning process and subsequently eliminated to get enlarged pore size. This innovative method was used to subdue two weaknesses of PCL electrospun fibers which are small pore size and lack of bio-inductive property. The scaffolds with improved pore diameter and interconnectivity enabled enhanced cell infiltration and homogeneous cell distribution, thus creating the potential to generate functional tissue [182].

An electrospun composite containing uniformly distributed but distinct fibers of PCL and PEO was developed. In this composite scaffold, fibers of polyethylene oxide formed the removable sacrificial fiber fraction. Both polymers were chosen based on their stability in hydrated environment, such as PCL is slowly degrading, whereas PEO dissolves immediately upon hydration. Although, removal of sacrificial fiber content resulted in reduced mechanical properties, it increased the size of the pores within the scaffold. Increase in sacrificial fiber fraction in the construct augmented cellular infiltration within construct. Construct with 60% PEO fraction, was observed to be fully colonized with seeded cells and was able to direct cell morphology and consequent matrix formation [183].

### 6.4. Bone Tissue Regeneration

Bone is one of the highly vascularized tissues in the human body. It is categorized into cortical bone and trabecular bone. Cortical bone is a dense, solid bone, extends mechanical support to human body and protection to bone marrow whereas trabecular bone is biologically active, enables joint and limb movement. The bone structure is made up of 69% of inorganic component containing hydroxyapatite and calcium phosphate complex contributing to bone its compactness and stiffness, while organic component composed of collagen and other structural proteins accounts for about 22% [184].

The regeneration of bones is a complex process involving a series of osteoinductive processes. Therefore bone TE demands the utilization of scaffolding, cells, chemical signaling and mechanical forces to create customized tissues. Biomimetic scaffolding for bone repair can include features such as high porosity to aid cell attachment, migration, proliferation and differentiation and biomechanical stress tolerance ability to endure stress generated within body during tissue regeneration [60]. A growing number of bone illnesses including infections, cancer and bone loss, necessitate bone regeneration. The vigorous course of bone TE begins with movement and recruitment of osteoprogenitor cells, and continues with cellular growth, differentiation, matrix production, and bone remodelling. Mechanical characteristics of bones are due to unique structural design of bone that extends from nano range to macro range dimensions, along with specific interconnections. Bone TE focuses on developing three-dimensional scaffolds that can replicate the ECM, offer structural support as well as aid in regeneration of bone. To increase the attachment, viability and mobility of osteogenic cells, scaffolds should have osteo-conductive, osteo-inductive, and osteogenic characteristics. To impart these characteristics, scaffolds are manufactured to provide mechanical and chemical cues that induce osteoblastic lineage formation [185].

Several scientists have attempted to alter the mechanical properties of scaffolds namely stiffness, strength and toughness using various methods and to create nanostructures to imitate bone’s natural architecture [186]. Despite many studies focusing on bone TE, much-needed advancements in scaffolds with conceivably superior clinical outcomes are still required. Electrospinning has long been considered an appropriate manufacturing technique for scaffolds by virtue of its multidimensional capacity of making nano- and micro-range fibrous frameworks with configurable fiber features [187].

Using an electrospinning process, PLA fibers encapsulating Fe_3_O_4_ nanoparticles at concentration of 2 and 5 percent were formed. Bone deformities transplanted with Fe_3_O_4_/PLA nanofibers displayed a significantly greater rate of bone healing compared to deformities transplanted with plain PLA nanofibers. Furthermore, CT scan demonstrated that the bone defects grafted with Fe_3_O_4_/PLA nanofibers encapsulating 2 and 5 percent Fe_3_O_4_ nanoparticles presented 1.9- and 2.3-fold enhancement, respectively, in volume of bone in comparison to the control sample [188]. Miszuket al. fabricated a composite nanofiber based scaffold using polycaprolactone/hydroxyapatite for regeneration of bone using an new thermally induced self-agglomeration (TISA) technique based on electrospinning. High elasticity, porosity even after coating with minerals and easy alteration with the applied pressure to fit to different defect shapes are the reported features making it desirable for application in bone TE. In addition, biomimetic mineral coating on fabricated scaffolds allows simultaneously encapsulation of different types of proteins, small molecules and drugs, under physiologically mild conditions. This study suggested that the innovative nanofiber based composite scaffold, that are press-fit, can be a sound means to deliver multiple drugs along with bone TE [189].

### 6.5. Dermis Tissue Regeneration

Skin lesions usually heal by forming epithelialized scar tissue rather than full skin regeneration. The epidermis has a poor ability to heal compared to the dermis; thus in case of substantial damage to the epidermis, biological regeneration process is insufficient. On the other hand, the dermis has a tremendous ability to rejuvenate. After a skin injury, the scar tissue develops with deficiency of dermis, thus loses the flexibility, elasticity, and toughness of natural dermis [190]. The fibrous structure in native ECM always shows a more intricate design than just straightforward unidirectional alignment. Skin tissue contains collagen fibrils that have a pattern like a basketweave or mesh. As a result, scaffolds with crossed nanofibers performed better than those with random or unidirectionally aligned nanofibers in terms of keratinocyte and fibroblast migration rates.

Collagen in its indigenous form acts as a natural foundation for cell adhesion, division, growth and specialization. Collagen exhibits significant strength in its biological form [191]. In addition, its’ biological origin make it the most biomimetic skin substitute created and thus the most preferred material to fabricate nanofibrous scaffold.

Powell and Boyce prepared electrospun submicron fibers using PCL and collagen to design a scaffold. Mechanical performance of nanofibers improved noticeably with mixing of little amount of PCL to collagen without negotiating on the biocompatibility of nanofibers. Keratinocytes and dermal fibroblasts cultured on collagen/PCL nanofiber scaffolds promoted the regeneration of the layered epidermis, dermis and uninterrupted basal layers [192]. Another study intended to evaluate in vivo performance of the PGA/collagen nanofiber on granulation histology and its capability of stimulating new vasculature was conducted out by Sekiya et al. This group of researchers developed PGA/collagen nanofibers using electrospinning technique. When compared to commercially available collagen matrix, histology revealed that fabricated nanofibers demonstrated considerably higher cell density with greater number of migrating cells. These observations indicated the superior ability of the developed nanofibers in relation to cell migration and neovascularization compared to collagen matrix product. This desirable outcome was attributed to the nano-range diameter of fibers and inclusion of PGA [193]. In another study, a highly porous scaffold created out of PCL/chitosan fibers with core–shell morphology were developed using emulsion electrospinning. Presence of high porosity and interconnectivity assisted penetration and proliferation of cells. The scaffold also supported ECM protein translation and in vitro layered epithelialization. Successful incorporation of the scaffold with margins of wound in animal model and rapid healing in around 20 days established the effectiveness of the scaffold as skin graft [194].

### 6.6. Cardiac Tissue Regeneration

Cardiac tissue has very restricted regenerative capacity, thus cardiac tissue regeneration using the principles of TE is a necessary and appropriate alternative. Some of the challenges associated with cardiac tissue engineering are the choice of polymers for fabricating scaffolds and achieving the required alignment of the microfibrils for guiding the growth of cells and contraction of cardiac muscle cells. Another clinical challenge is the regeneration of heart valves (HVs) because of their complex anatomical structure with leaflets and numerous supporting structures along with having complex, striated ECM [195]. Earlier many attempts to engineer the valves met the failure with disordered ECM and inability to function due to use of isotropic and homogeneous scaffolds [196]. Biomimetic scaffold with heterogeneous and anisotropic characteristics which approach that of inherent heart valve tissue are applicable for regenerating HV tissue. Tissue engineered HVs are contemplated to be capable of adapting to such complexities, indicating its potential as a alternative to existing treatments.

Ahmadi et al. manufactured polyurethane/chitosan/carbon nanotubes (PU/Cs/CNT) composite nanofibrous scaffolds using two techniques. PU/Cs/CNT electrospun scaffolds were manufactured by blending CNT and electrospinning this blend of polyurathane, chitosan and CNT. In other technique, polyurethane/chitosan solution electrospun into nanofibers and CNT were electrosprayed onto nanofibers from the opposite side. The nanofibers were also collected with random and aligned orientation. Addition of CNT substantiallyameliorated the mechanical characteristicsand hydrophilicity of the nanofibers. Improvement of surface properties by hydrophilic chitosan and carboxylated CNTs led to proliferation enhancement of Human umbilical vein endothelial cells in PU/Cs/CNT scaffold compared to PU scaffold. Cardiac rat myoblast cells (H9C2 cells) proliferation on fibrous matrix with electrosprayed CNT was more notable than cell proliferation on PU scaffold. Alamar blue assays demonstrated that number of H9C2 cells on scaffold with electrosprayed CNT, in both aligned and random scaffolds, enhanced notably higher than other scaffolds and control group [197].

To fabricate fibrous scaffold for replicating the anisotropic nature of native heart valves, Xue et al. utilised ring-shaped copper collector for collecting electrospun fibers. This group of researchers fabricated anisotropic fibrous matrices manufactured with (poly(1,3-diamino-2-hydroxypropane-co-glycerol sebacate)-co-poly (ethylene glycol) (APS-co-PEG) and PCL polymer blends that hadadjustable and controllable fiber morphologies and mechanical features. The polymer formulationswere electrospun onto flat aluminum foil and ring-shaped copper wire, producing isotropic and anisotropic fibers, respectively. The scaffolds gathered on flat aluminum foil demonstratedalike mechanical properties in the two perpendicular directions, revealing an isotropic behavior, whereas the scaffolds collected on the ring-shaped collector acted differently in their fiber and cross-fiber directions, indicating mechanical anisotropy. The anisotropic scaffold also showed to possess a Degree of anisotropy (DA) close to that of a porcine aortic valve, indicating its prospectives to be used to regenerate the heart valves [198].

## 7. Conclusions

We can witness the tremendous work done in the discipline of TERM where nanofibrous scaffolds have been employed as reinforcement to allow regeneration of tissue. Promising outcomes of the TERM research conducted for the variety of tissues and several disorders is increasing hopes for a therapy that will be a better alternative to existing therapies. Among the available therapies, graft surgery is indispensible for numerous health issues. Extensive research in TERM is taking the discipline forward by small but consistent leaps. Kind of physical and chemical cues, their amount and timing for the regulation of cell activities has still puzzled the researchers. Increasing comprehension about the cellular behavior to an array of physical and chemical cues will help the researchers to come up with the right approach for tissue regeneration.

Natural and synthetic polymers have their own set of advantages which it offers to the fabricated scaffolds for the tissue regeneration purpose. Natural polymers offers features such as biocompatibility, biodegradability, low immunogenicity and its ability to elicit the favorable interaction with the cells, whereas synthetic polymers offers characteristics of their fitness to spinning, excellent mechanical strength and cost-efficiency. Features of both natural and synthetic polymers are complementary too each other explaining their often use in combination to bring desirable attributes in the scaffold. Nonetheless, further research in the field will need to focus on innovative polymers with characteristic features for specific tissue regeneration applications.

Electrospun nanofibers have been integrated with 3D printed tissue constructs fabricated using additive manufacturing or 3D printing technique, which is another promising technology, to design composite scaffolds. The merit provided by the nanofibers was the enhancement in infiltration of the cells in the scaffold, whereas 3D printed component of the composite extends its mechanical strength to the scaffold and also enables preparation of complex 3D forms at the macro level. Such alliances between the matrices are significant for regeneration of load bearing tissues requiring mechanical strength of the scaffold such as bone and cartilages. Similarly electrospun nanofibers composites with hydrogels have been experimented to overcome limitation of both of them and to reap the merits provided by both components for the TE purpose.Such combination can be studied more for the purpose of soft tissue regeneration. More of such combinations involving more than one matrix fabricated using different techniques needs to be studied to mimic the biological ECM in various tissues to a maximum extent.

To achieve high scale production of nanofibers is still challenging due to the low yields of existing processes. Centrifugal jet spinning has shown to be capable of producing high quantities of nanofibers in short duration and consuming less power. This technique also has the potential for scaling up to commercial production levels [199,200]. Other unattended challenges associated with existing nanofiber fabrication methodsinclude utilization of toxic solvents and low and uncertain cell penetration into fiber matrices. High cost of the biomedical research also adds to existing obstacles in the path of researchwhich needs to be addressed to reach the feasible as well as affordable solution.Given the potential demonstrated by Nanofibers in TERM, new pursuits in the application of nanofibers in TERM are anticipated to deepen understanding of tissue regeneration, to bring answers to unsolved queries and to offer intervention to apply on wide population to regenerate tissues.

## Figures and Tables

**Table 1:** Advantages and disadvantages of natural and synthetic polymers.

	Advantages	Disadvantages
Natural polymers	Inherently bioactive Possess cell-interactive groups on their backbones Offer better cell attachment, growth, multiplication and differentiation Chemically benign degradation products Elicit low immune response	Difficult processing Low cost effectiveness Poor mechanical properties Precarious outcome due to batch-to-batch variations Insufficient mechanical strength Hydrophilicity Need of crosslinking to improve strength
Synthetic polymers	High flexibility in the processing More economical Tunable mechanical properties Higher mechanical strength Better structural stability	Lacking bioactivity May produce intense immune response Necessitate more modifications compared to natural polymers to impart bioactivity

**Table 2:** Natural and synthetic polymers used for tissue engineering applications.

Polymer/Polymer Composite	Novel Step	Electrospinning Technique	Application	Result
Collagen nanofibers [19]	Electrospun nanofibers were treated with catecholamines and calcium choride followed by exposure to ammonium carbonate to enable the formation of in situ crosslinked collagen-CaCO_3_ composite scaffolds.	Electrospinning	Bone tissue engineering	Inclusion of Ca^2+^ into catecholamines containing collagen and ensuing mineralization improved the elastic features, mechanical strength and stiffness. Human Fetal Osteoblasts demonstrated enhanced cell proliferation and osteogenic differentiation in the mineralized composite mats compared to pristine collagen mats.
Gelatin nanofibers [20]	Mild solvents have been utilised to preserve gelatin in a sol state at ambient temperature, for the electrospinning of nanofibers. A model protein reagent, (ALP) was embedded in the gelatin nanofibers to evaluate protein stability	Single nozzle electrospinning	Tissue engineering scaffolds	Mild neutral dipolar aprotic solvents, N,N-dimethylacetamide (DMA), N,N-dimethylformamide (DMF) and N-methyl-2-pyrrolidone (NMP), allowed gelatin to remain in sol state at room temperature. DMA, DMF and NMP conserved the alkaline phosphatase activity substantially, indicating their effectiveness for encapsulating protein reagents while preserving their activities. Swiss 3T3 fibroblasts grew well on the manufactured gelatin nanofibers.
Sodium alginate/polycaprolactone core-shell nanofibers [21]	Emulsion electrospinning of sodium alginate has been tried to fabricate nanofibers with core-shell morphology	Water-in-oil emulsion electrospinning	Tissue engineering scaffolds and controlled drug delivery	Increase in PCL concentration improved the loss and storage moduli and also increases the diameter of the manufactured fibers. Cytotoxicity assay using human dermalfibroblasts indicated no cytotoxicity of the manufactured core-shell nanofibers.
Chitosan/hydroxyapatite (HA) nanofibers [22]	HA nanopowder was dispersed in chitosan solution to be electrospun to replicate the structure and composition of natural bone tissue. Cross-linking was carried out with exposure to the vapors of a glutaraldehyde	Blend electrospinning	Bone tissue engineering	Addition of HA caused statistically significant reduction in the average fiber diameter and an enhancement in Young’s modulus and Ultimate Tensile Strength compared to chitosan nanofiber samples. High cell viability was observed for HA incorporated chitosan nanofibers.
PVA/Hyaluronic acid nanofibers [23]	Incorporated cellulose nanocrystals (CNCs) as nanofiller to improve mechanical properties of the nanofibers. L-arginine was loaded as wound healing accelerator.	Blend electrospinning	Dermal tissue engineering	Inclusion of CNCs into PVA/HA blend substantially augmented mechanical and swelling properties of nanofibers. PVA/HA/CNC/L -arginine nanofibers displayed excellent hemocompatibility, enhanced protein adsorption, remarkable proliferative and adhesive capability.
SF/kappa-carrageenan nanofibers [24]	kappa-carrageenan was blended with SF for electrospinning nanofibers to improve biological properties of SF based nanofibers and to mimic bone ECM structure, while genipin was used for crosslinking agent.	Blend electrospinning	Bone tissue engineering	Blending of kappa-carrageenan in nanofibrous matrix effectively moderated the hydrophobic nature of SF nanofibers, thus enhancing cell survival and proliferation. The scaffold was able to guide the osteogenic differentiation, stimulate mineralization and developement of bone tissue in vitro. Ultimate tensile strength and Young’s modulus of the SF mats improved post-crosslinking with genipin.
Poly caprolactone (PCL) electrospun nanofiber [25]	PCL electrospun nanofibrous matrix was combined with hydrogels of polyethylene glycol diacrylate (PEGDA), sodium alginate (SA) and type I collagen (CG1) to fabricate three kinds of scaffolds. Composite scaffold were created using the layers of hydrogel and PCL nanofibers.	Electrospinning	Dermal tissue engineering	Cells were more capable of proliferating and differentiating in the CG1-PCL scaffold compared to PEGDA-PCL and SA-PCL. The mean number of cells proliferated was greater for the CG1-PCL scaffold in comparison to other scaffolds. CG1-PCL also has lower hydrophilicity and degradability compared to PEGDA-PCL and SA-PCL which makes it appropriate as a dermal equivalent.
Polyaniline-co-(polydopamine grafted-poly(D,L-lactide) [PANI-co-(PDA-g-PLA)] electrospun nanofibers [26]	PANI-co-PDA was manufactured using a single -step chemical oxidization approach. Later, D,L-lactide monomer was inserted onto PDA segment using a ring opening polymerization to create PANI-co-(PDA-g-PLA) terpolymer. PANI and PDA were incorporated to improve hydrophobicity and biological activity of PLA.Fabricated terpolymer was electrospun into nanofibers and a conductive nanofibrous matrix was fabricated.	Electrospinning	Bone tissue engineering	The surface wettability of the scaffold was found acceptable for a successful TE application. Manufactured scaffold demonstrated exceptional performance in terms of adhesion, migration and proliferation of the mouse osteoblast MC3T3-E1 cells, primarily because of excellent and accessible binding cites in the scaffold owing to presence of PDA and PLA chains, biocompatible nature of PANI-co-(PDA-g-PLA) nanofibers and communication between the cells via electrical conductive matrix.
Polyglycolic acid/gelatin nanofibers [27]	Blend of Gelatin with PGA was electrospun into nanofibers. The polymer blend was utilised to enhance cell attachment, improve survival of the cells of the vasculature, namely endothelial and smooth muscle cells, and to impart appropriate biomechanical properties to the scaffold. Variable weight proportions of gelatin was tried to fabricate electrospun fibrous scaffolds.	Blend electrospinning	Vascular tissue engineering	Incorporation of gelatin substantially improved tensile strength and the Young’s modulus of the fiber sheets. Electrospun fibers with PGA and 10 wt% and 30 wt% gelatin had tensile strength values approximating that of natural vein values.Fibers with PGA and 10 wt% gelatin showed enhanced endothelial cells density whilst PGA with 30 wt% gelatin increased smooth muscle cell density with enhanced adhesion and survival compared to other scaffold blends.
Polyvinyl alcohol (PVA) electrospun nanofibers [28]	Epidermal growth factor (EGF) and fibroblast growth factor (FGF) were included into PVA to be co-electrospun into nanofibers for the fabrication of wound dressing. Single, mix, multilayer electrospun nanofibers were fabricated.	Electrospinning	Dermal tissue engineering	Fiber diameter decreased, surface roughness decreased, wettability increased after incorporation of growth factors within the PVA Nanofibers. The GFs incorporation in PVA nanofibers induced cell proliferation and better cell attachemnt compared to PVA control sample. PVA-growth factors nanofibrous matrix demonstrated to be a better scaffold to heal burn-wounds in comparison to PVA only nanofiber.
Dipeptide polyphosphazene-polyester blend nanofibers [29]	Polymeric blend composed of poly[(glycine ethyl glycinato)_1_ (phenylphenoxy)_1_ phosphazene] (PPHOS) and poly(lactide-co-glycolide) (PLAGA) in a 25:75 weight ratio was chosen to fabricate the BLEND nanofi bers via electrospinning. Biomimetic scaffolds were fabricated with concentric orientation of fibers with an open central lumen to mimic bone marrow cavity, as well as the lamellar structure of bone.	Electrospinning	Bone tissue engineering	The tensile strength value for BLEND nanofi bers was 25% higher than the tensile strength of trabecular bone. BLEND nanofiber matrices assisted osteoblasts attachement and proliferation and demonstrated an enhanced phenotype expression compared to polyester nanofibers. Additionally, the 3D structure supported osteoblast infiltration and ECM secretion, bridging the spaces in concentric walls in scaffold during in vitro culture. Scaffolds showed similar lamellar ECM organization to that of native bone

**Table 3:** Functionalization with bioactive molecules and their applications in tissue engineering.

Bioactive Molecule	Method of Functionalization	Research/Study	Outcome of Biofunctionalization	Cells Used/Tissue to Regenerate
Collagen [94]	Remote plasma treatment followed by immobilization of collagen on the nanofibersurface	PCL nanofibers were electrospun and layered with collagen	Collagen coating improved hydrophilicity and increased cell proliferation compared to non-coated PCL nanofibers	Primary human dermal fibroblasts (HDFs)/ Dermal tissue
Collagen [95]	Coaxial electrospinning technique and by soaking the PCL matrix in collagen solution	PCL nanofibers were electrospun and coated with collagen using two techniques	Density of human dermal fibroblasts on collagen layered PCL nanofibers prepared using coaxial electrospinning increased linearly compared to roughly collagen coated and uncoated PCL nanofibers	Human dermal fibroblasts/Dermal tissue
Gelatin [96]	Air plasma treatment followed by covalent grafting of gelatin molecules	PCL nanofibers were electrospun and grafted with gelatin molecules	Viability and proliferation rate of fibroblast cells increased in biofunctionalized nanofibers compared to tissue culture polystyrene (TCPS)	Fibroblast cells/Tissue engineering
Fibronectin [97]	Three different approaches were used -protein surface entrapment, chemical functionalization and coaxial electrospinning	PCL nanofibers were electrospun and functionalized with fibronectin using three approaches	Improved cell adhesion and proliferation of bone murine stromal cells was observed for scaffolds functionalized using all the three approaches, but sample with the surface entrapment of fibronectin demonstrated better performance.	Bone murine stromal cells/ bone tissue
Fibronectin [98]	Immersing in fibronectin solution overnight.	PCL nanofibers were electrospun with radial alignment and coated with fibronectin	Improved cell adhesion, cell migration and helped in more uniform distribution of cells. Boosted the effect of topographic cues offered by the fiber alignment.	Dural fibroblast cells/dural tissue
RGD [99]	RGD peptide was conjugated on nanofibers using Polyethylene glycol as a spacer.	Polyurethane electrospun matrix was immobilized with RGD peptide.	Improved viability, promoted proliferation of cells in comparison with an unaltered surface.	Human umbilical vein endothelial cells/vascular tissue
RGD [100]	RGD functionalization via strain-promoted azide–alkyne cycloaddition.	PCL aligned nanofibers were electrospun and functionalized with RGD peptide.	RGD functionalization decreased muscular atrophy and hastened sensory recovery. Facilitated regeneration of sciatic nerve in animal model compared to non-functionalized nanofibers.	Rat sciatic nerve repair
RGD [101]	Chemical conjugation of RGD on nanofibers was carried out, after activation of carboxyl groups of polymer	Polybutylene adipate-*co*-terephthalate (PBAT)/gelatin elctrospun nanofibers were loaded with Doxycycline and modified using RGD	RGD functionalized PBAT/gelatin nanofibers showed notably improved wound closure and histopathological results with re-epithelialization and angiogenesis in animal model compared to the control groups.	Dermal wounds
Aspartic acid (ASP) and Glutamic acid (GLU) Templated Peptides [102]	Cold atmospheric plasma (CAP) was used to modify the nanofiber surface and to mediate the conjugation with peptides	PLGA nanofiberswere electrospun and conjugated with peptides	Peptide conjugation improved the osteoinductive capacity of nanofibers. ASP templated peptide conjugation to nanofibers increased the expression of key osteogenic markers and induced cell proliferation more than GLU templated peptide conjugated nanofibers.	Human bone marrow derived mesenchymal stem cells/bone tissue
Laminin [103]	Physical coating method and the chemical bonding method used for functionalization of the surfaceof the nanofiber	Slow-degrading silica nanofibers were electrospun and attached with Laminin on the surface	Nanofibers with covalently attached laminin showed significantly longer neurite extensions than those observed on unmodified nanofibers and nanofibers subjected to physical adsorption of laminin.	Rat pheochromocytoma cell line/neuron
Laminin [104]	covalent binding, physical adsorption or blended electrospinning procedures.	PLLA nanofibers were electrospun and modified with laminin.	Functionalized nanofibers were capable of enhancing axonal extensions. In comparison to covalent immobilized and physical adsorbed, blending for electrospinning of laminin and synthetic polymer is a simple and effective method to functionalize nanofibers	Rat pheochromocytoma cell-line PC12 cells/neurons
Laminin [105]	Functionalization with laminin usingcarbodiimide based crosslinking and physical adsorption method	Nanofibers were electrospun from the blends of poly(caprolactone) (PCL) and chitosan and modified with laminin	Number of cells attached and the rate of proliferation on the laminincoated scaffolds were higher than those of pure PCL and PCL-chitosan scaffolds. Schwann Cell attachment and proliferation were significantly higher on PCL-chitosan scaffolds with crosslinked laminin than the PCL-chitosan nanofibrous matrices with adsorbed laminin.	Schwann Cell/nerve tissue
Avidin-biotin system [106]	Avidin immobilization on nanofibers	Poly(caprolactone-co-lactide)/Pluronic (PLCL/Pluronic) nanofibers were electrospun and modified with avidin. Adipose-derived stem cells (ADSCs) were modified with biotin.	Biotinylated ADSCs showed more rapid attachment onto avidin-treated nanofibrous matrices compared to normal ADSCs adherence on untreated matrices, and the difference of attached cell number between the two groups was notable. It also promoted cell spreading on nanofibrous matrices.	Adipose-derived stem cells (ADSCs)
Fibroblast Growth Factor-2 (FGF-2) [107]	FGF-2 was immobilized on the surface of the nanofibers through avidin-biotin covalent binding.	Gelatin nanofibers were electrospun, crosslinked using glutaraldehyde, and modified with FGF-2	FGF-2 immobilization led to proportionate increase in cell proliferation and adhesion.	Adipose derived stem cells
Insulin [108]	Insulin was bound to carboxylic moieties of the polymer backbone through a standard carbodiimide chemistry	PCL and cellulose acetate micro-nanofibers were electrospun and functionalized with insulin.	Enhanced expression of tendon phenotypic markers by Mesenchymal stem cells (MSCs) akin to observations from insulin supplemented media, indicatedconservation of insulin bioactivity upon functionalization.	MSCs/tendon
Insulin-like Growth Factor-1 (IGF-1) [109]	Physical adsorption of IGF-1 due to soaking into suspension of IGF-1 in PBS and shaking for 4 h	Graphene oxide (GO)-incorporated PLGAnanofibres were electrospun and functionalized with IGF-1	Survival, proliferation, and differentiation of neural stem cells (NSCs) was significantly increased. Higher survival rate of NSCs in the IGF-1 modifed nanofibers compared to unmodifed nanofibers was observed.	NSCs/nerve cells
Polydopamine assisted bromelain [110]	Soaking in solution of dopamine and bromelain, with continuoue stirring for 8 h. Dopamine-assisted co-deposition strategy was used.	PCL nanofibers were electrospun and immobilized with bromelain using polydopamine (PDA) to create bromelain-polydopamine-PCL (BrPDA-PCL) nanofibers	BrPDA-PCL fibers exhibited superior biocompatibility compared to PCL fibers PDA coating made scaffold hydrophilic, allowing for better cell attachment and spreading PDA and bromelain both showed anti-bacterial activity.	L929 fibroblast cells/wound healing
Poly norepinephrine (pNE) [111]	Soaking in norepinephrine solution for 15 h	PCLfibers were electrospun andcoated using mussel-inspired pNE.	pNE coating improved the ECM proteins accumulation on the fibers, which supported cell adhesion and proliferation of cells on PCL fibrous membranes.	Skeletal muscle cell line L6/skeletal muscles
pNE mediated collagen [112]	Soaking in norepinephrine solution 16 h, followed by soaking in collagen solution overnight.	Poly(lactic acid-co-caprolactone) (PLCL) nanofibers were electrospun and coated with poly norepinephrine, followed by collagen.	pNE coating assisted in collagen anchoring to improve cell adhesion and to immobilize nerve growth factor to advance differentiation to neurons. pNE–collagen coating was observed to be the better substrate for PC12 cells differentiation.	PC12 cells/neurons
Polyphenol [113]	Blend electrospinning	Polylactic acid/date palm polyphenol nanofibers were electrospun using blend electrospinning.	Both cell proliferation and cell viability were enhanced with increased polyphenol concentration within the scaffolds. Higher polyphenol content resulted into better cell migration	NIH/3T3 fibroblast cell/wound healing
Vascular endothelial growth factor (VEGF) [114]	Blend and co-axial electrospinning	PCL-gelatin nanofibers were electrospun and modified with VEGF.	Functionalization improved proliferation of mesenchymal stem cells, but no significant difference in proliferartion between nanofibers manufactured with both techniques was observed. Expression of cardiac specific proteins enhanced.	Human mesenchymal stem cells/myocardium
VEGF [115]	Covalent coupling to VEGF by forming stable amide bond	PCL nanofibers were electrospun and modified with VEGF.	Biological activity of immobilised VEGF was maintained and functionalised substrates demonstrated to induce a higher cell number compared to non-functionalised scaffolds.	Human umbilical vein endothelial cells
Epidermal growth factor (EGF) and fibroblast growth factor (FGF) [28]	Blend electrospinning	PVAnanofibers were electrospun and modified with EGF and FGF.	GFs incorporated PVA nanofibers induced cell proliferation andenhanced cell survival compared to PVA control sample In in-vivo study, PVA/EGF/FGF nanofibers demonstratedquick recovery of the wounds in contrast to that of only EGF or FGF nanofibers.	Human dermal fibroblasts/wound healing.

**Table 4:** Different electrospinning techniques to develop Nanofibers—Their Advantages and Limitations.

Electrospinning Technique	Advantages	Limitations
Single nozzle electrospinning	Simple process with least number of controllable parameters Most experimented due to simple gear and process used	Compatibility between polymer/polymer solutions/bioactive molecules is essential to eject mixture through single nozzle. Involves the use of solvents For electrospinning of polymer blend, availability of common solvent is essential Lower rate of production Denaturation of sensitive bioactive agents or loss of their bioactivity due to presence of the solvents, in case of blend of bioactive with polymer(s)
Co-axial electrospinning	Actives which are lacking fibrous characteristics can still be enclosed in the nanofiber core. Two or more bioactive compounds with varied biological activity and solubilities can be encapsulated within single multilayered fiber. Controlled drug release is possible due to core and shell morphology	Sophisticated gear required Use of co-axial needle make electrospinning process complex, involving numerous controllable parameters
Multi-nozzle electrospinning	Increased production rate Multiple nozzles can be arranged in various configurations to alter alignment of nanofibers	Increases complications in process with repulsion between jets, non-uniformity of electrical fields, poor control over the process and decline in fiber quality
Emulsion electrospinning	Able to fabricate core-shell Nanofibers without use of co-axial nozzle Hydrophilic or hydrophobic actives can be loaded into core of nanofibers by electrospinning W/O or O/W emulsions respectively	Formulation of emulsion eliminates the requirement of common solvent for polymer and bioactive molecules
Cell electrospinning	It includes all the advantages of electrospinning In addition, it is capable of embedding living cells inside the fibers encapsulation of cells within fibers enables effective and rapid exchange of nutrients and oxygen enables excellent interaction between cells and help to achieve uniform cell distribution	More critical variables to consider to keep cells viable in electrospun fibers Low mechanical properties Low control over cell density

## Data Availability

Data sharing is not applicable to this article.
